# A multi-centred pilot randomised controlled trial of learning through play plus culturally adapted cognitive behaviour therapy for treating postnatal depression in Nigerian women

**DOI:** 10.3389/fpsyt.2025.1552406

**Published:** 2025-05-05

**Authors:** Dung Ezekiel Jidong, Tarela Juliet Ike, Maigari Yusuf Taru, Juliet Yop Pwajok, Charles Nnaemeka Nwoga, John Ezekiel Jidong, Christopher Francis, Shadrack Bitrus Mwankon, Karick Haruna, Zubairu Dagona, Nusrat Husain

**Affiliations:** ^1^ Division of Psychology and Mental Health, University of Manchester, Manchester, United Kingdom; ^2^ Global Centre for Research on Mental Health Inequalities, Mersey Care NHS Foundation Trust, Liverpool, United Kingdom; ^3^ Department of Sociology and Criminology, Teesside University, Middlesbrough, United Kingdom; ^4^ Department of Psychiatry, Jos University Teaching Hospital, Jos, Nigeria; ^5^ Department of Psychology, University of Jos, Jos, Nigeria

**Keywords:** anxiety, culturally adapted, intervention, postnatal depression, mothers, Nigeria

## Abstract

**Background:**

About 13% of women globally experience postnatal depression with adverse implications for the mothers and their children. In Nigeria, there is limited access to evidence-based culturally appropriate care for mothers affected by postnatal depression.

**Methods:**

This study was a multi-centre, two-arm, parallel-group, single-blind, individually randomised controlled trial design adopted to test the feasibility, cultural appropriateness and acceptability of Learning Through Play plus Culturally adapted Cognitive Behaviour Therapy (LTP+CaCBT). The LTP+CaCBT is a 12-session (90 minutes each) intervention to treat postnatal depression, and this was compared with the Enhanced Treatment As Usual (ETAU). Sixty-six mother-child pairs across three centres who scored >5 on the Patient Health Questionnaire (PHQ-9) were recruited for the study and randomised to either the LTP+CaCBT experimental or ETAU control groups. Data were collected at various time points (baseline, end of intervention and 3 months post-enrolment) and analysed using appropriate descriptive and inferential statistics. N = 3 focus groups comprising 11 participants each and n = 18 individual interviews were conducted to explore participants’ experiences engaging with the intervention. Interviews were transcribed verbatim and analysed using interpretative phenomenological analysis.

**Result:**

The LTP+CaCBT group (n=33) recorded a high participants’ recruitment, participation and retention rate of 94% across 12 sessions. Satisfaction with intervention (LTP+CaCBT, 97%; ETAU, 34.4%). reduction in postnatal depression was higher in LTP+CaCBT on PHQ-9 *Md* = 3.00 with z= -4.935; compared to ETAU, *Md*=4.00 with z= -2.556. Improvement was also recorded for the anxiety and social support level; there was no improvement for the control group, as the scores remained the same. Themes identified from the qualitative dataset showed positive behaviour management, enhanced mother-child interaction and relationship, modification of negative thought processes, positive experience and relationship formation.

**Conclusion:**

The LTP+CaCBT intervention is shown to be acceptable and culturally appropriate whilst indicating potential clinical effectiveness in reducing postnatal depression and anxiety in Nigerian mothers. A fully powered RCT is recommended to evaluate the clinical and cost-effectiveness of LTP+CaCBT, including the child’s outcomes compared with ETAU.

**Clinical trial registration:**

https://clinicaltrials.gov/study/NCT04644081, identifier NCT04644081.

## Introduction

The prevalence of postnatal mental health conditions like depression and anxiety is estimated to affect 80 million women globally, corresponding to a burden of disease equivalent to over 800,000 years of life lived with disability in women (1). A meta-synthesis of 14 empirical studies found 210 per 100,000 cases of depression and suicidal attempts occurred during postnatal periods, with long-term negative consequences on their children (2, 3). This is more prevalent in low- and middle-income countries (LMICs). There are over 250 million children who lack adequate developmental support in LMICs, including Nigeria, due to mothers’ postnatal depression, and these children are unable to reach their full potential in life (4).

The World Health Organisation (WHO) (5) data suggest that 13% of women who have just given birth experience depression. In developing countries like Nigeria, this is even higher, with 19.8% of women suffering from depression after childbirth (6). In Nigeria, postnatal depression is prevalent in the northern region, experiencing the highest at 44.5% (7). Several factors have been attributed to the high rate of postnatal depression, including socioeconomic status, unemployment, education and religion (8, 9), including other factors associated with the cultural preference of a male child (10). Other studies have also attributed factors such as previous history of depression, poor social support stress, and adverse relations between the mother and her partner as significant predictors and contributors to postnatal depression (11).

The effect of postnatal depression could have a profound effect on the mother and child’s well-being, and this includes poor hygiene of the others and child welfare, risk of infanticide, suicidal ideation, poor mother-child attachment, infant care and breastfeeding. Within the context of Nigeria, Jidong et al. (12) study of 40 mothers showed that mothers experienced persistent psychological distress from labour to birth and experienced cultural practices that impacted their feelings. It was also found that the mother had poor knowledge about childcare, access to support, and healthy nutrition (12), which recommends knowledge-based interventions such as learning through play plus culturally adapted cognitive behaviour therapy for mothers suffering from postnatal depression.

Previous literature on intervention for postnatal depression has often tended to be western dominated and focused on exercise-based interventions (13, 14), digital health intervention (15), midwife-led breastfeeding group intervention (16) and online peer-delivered group cognitive-behavioural therapy (5). However, there remains a gap in examining the culturally appropriate, feasible and acceptable of LTP+CaCBT amongst postnatally depressed mothers in Nigeria. However, some previous studies that have tested LTP+CaCBT with a focus on British African and Caribbean populations in the UK (17, 18), and mothers in Kilifi, Kenya (19) and South Asian women (20). Nevertheless, there remains a gap in testing this intervention amongst depressed mothers in Nigeria. Studies have shown that access to mental health care in Nigeria is low due to a myriad of reasons, including lack of mental health awareness, poor mental health literacy, stigma, and socio-economic barriers, including lack of affordable mental health services (10, 21).

Our previous studies, including a systematic review of maternal health and child well-being in Nigeria (10) and a qualitative study of the maternal mental health lived experience of Nigerian mothers (12), are consistent that about 30% of Nigerian mothers suffer from postnatal depression (22). Yet, only one out of every five persons who suffer from mental health problems can access any care (23), depicting the treatment gap for postnatal depression. These gaps necessitated the current study, which makes an original and significant contribution by specifically seeking to address the cultural appropriateness and preliminary clinical effectiveness of LTP+CaCBT compared to ETAU for treating postnatal depression among mothers in Nigeria.

The Learning through Play (LTP) programme is a parenting intervention initially developed in Toronto, Canada, to train parents for their children’s healthy upbringing. The integrated CBT component is designed to encourage healthy thinking and behaviour modification. While the combined fusion of the LTP+CaCBT has been tested in other countries, such as South Asian (20, 24), the UK’s Africa and the Caribbean (17) and Kenyan (19) depressed women, we are unaware of the testing of this intervention in addressing postnatal depression in Nigeria. Hence, the research question below:

What is the cultural appropriateness, acceptability, feasibility and preliminary clinical effectiveness of Learning Through Play plus Culturally adapted Cognitive Behaviour Therapy (LTP+CaCBT) compared to Enhanced Treatment As Usual (ETAU) for treating postnatal depression among depressed mothers in Nigeria?

## Methods

### Design

This study is a multi-centre, two-arm, parallel-group, single-blind, individually randomised controlled trial design adopted to compare LTP+CaCBT with ETAU for treating postnatal depression in Nigeria. Due to its methodological rigour and replicability, RCTs are often considered the gold standard for testing psychological interventions (25).

### Ethics

The study received ethical approval (no. JUTH/DCS/IREC/127/XXXI/2579) from the Jos University Teaching Hospital Research Ethics Committee in Nigeria and an ethics exemption from the University of Manchester Ethics Committee (no. 2024-19993-34267).

### Recruitment

The research team recruited participants from the Jos University Teaching Hospital. We also leveraged our established networks and distributed printed copies of research flyers to places frequented by mothers in Jos and its environs, such as churches, mosques and community venues/organisations for mother-childcare. The inclusion criteria for participants to take part in the study comprised mothers from the trial catchment areas who are experiencing postnatal depression due to childbirth and/or parenting. For this study, postnatal depression is defined as the depression suffered by mothers of children between ages 0-3 years; residents in the trial catchment areas and are available for the study’s duration of 12 weeks and follow-ups at the end of the intervention and three months post-intervention; 18 years+ and able to give informed consent. The exclusion criteria included mothers who were below 18 years old, experiencing active suicidal ideation or any other severe disorders, and were unable to provide informed consent.

### Participants’ demography

In total, 66 study participants were recruited across three primary healthcare centres (n=22 participants each per centre). Of the 66 participants, two indicated having attained a primary level of education, whilst 38 indicated secondary education. The other 26 indicated tertiary-level education. Regarding employment, 24 were employed at the time of the intervention, whilst the other 42 were unemployed. For religious demography, 6 were Muslim, while 60 were Christians. The mothers’ age range was between 20 to 40 years. Their children’s age range was between 3 weeks for the youngest child and 17 months for the oldest.

### Randomisation

Microsoft Excel was used to randomise participants who met the study’s inclusion criteria into either the LTP+CaCBT or the ETAU group. Based on the list of eligible participants, we randomly generated the value of 0 or 1 using the RANDBETWEEN function in Excel, thereby choosing 0 and 1 as the range. All participants with 0 were assigned to the control (ETAU) group, and all participants with 1 to the experimental (LTP+CaCBT) group. The method of randomisation used enabled a single sequence of random assignments in which participants were assigned based on a 1:1 ratio (26). All participants had equal chances of receiving either the LTP+CaCBT or the ETAU intervention. All participants were blinded to whether they were assigned to experimental (LTP+CaCBT) or controlled (ETAU) groups.

### Assessments

The Patient Health Questionnaire (PHQ-9) was used to conduct assessment and screening for postnatal depression, and a Clinical Interview-Scheduled-Revised (CIS-R) was further administered to confirm the diagnosis of postnatal depression. In essence, participants who scored >5 and above on the PHQ-9 were recruited for baseline assessment and randomised into experimental (LTP+CaCBT) or controlled (ETAU) groups. A score of >5 on PHQ-9 is recommended to indicate postnatal depression in need of intervention (27).

The study’s primary outcome measures were to examine the cultural appropriateness, feasibility, and acceptability of the LTP+CaCBT intervention among depressed Nigerian women, which was assessed using the Service Satisfaction Scale (28). In terms of the secondary outcome measures, with assessments at baseline, 12 weeks and three months post-intervention, the scales used for assessment include the PHQ-9—a multipurpose instrument for screening, diagnosing, monitoring and assessing the severity of depression (29), the Generalised Anxiety Disorder Scale—a self-report questionnaire is employed to screen and evaluate symptom severity, with scores ranging from 0 to 21, where higher scores denote increased levels of anxiety (30) and Oslo Social Support Scale—a succinct, three-item self-reported measure designed to evaluate the extent of social support experienced by an individual. This measure emphasises close confidants, the presence of concern, and the individual’s relationship with neighbours (31). The above scales for assessing outcomes measures were strategically chosen for their flexibility and wide usage among Nigeria’s mental healthcare service providers.

### Cultural adaptation of LTP+CaCBT

An ‘Iterative Model of Co-adaptation’ (IMC) (17) for the LTP+CaCBT manual to ensure it is culturally appropriate and suitable for mothers with postnatal depression was adopted. The IMC process includes translation/co-adaptation of the manual via Focus Group Participatory Action Research (FG-PAR). It was also informed by a previous study with n=40 participants comprising mothers with lived experiences of postnatal depression taking cognisance of context-specific factors such as cultural and religious beliefs, language(s), literacy and preferences (12). All feedback as appropriate (i.e., exploratory study, seminars, conferences, and workshops) informs the manual’s clinical value, practical utility and how it could be integrated into local care systems. Our use of cultural adaptation is in line with previous literature, which emphasises its importance in ensuring the appropriateness of intervention (32–43). As Greene and Lee (32) argue, culture influences how individuals see themselves and interact with the environment. Emphasising the significance of cultural adaptation, Marsiglia et al. (43) contend that a culturally grounded approach to adaptation starts with assessing existing evidence-based interventions’ appropriateness and adapting as needed to ensure more relevance whilst engaging clients from various cultural backgrounds without compromising the intervention’s effectiveness. Perera et al. (33) model of cultural adaptation of low-intensity World Health Organisation Intervention emphasised four stages of adaptation, which include (i) gathering information on the characteristics of the local population, (ii) formulation of adaptation information based on the collated information, (iii) consultation with locals on the identified areas for adaption and iv) evaluations with local experts. This approach also informs our integration of cultural adaptation for the LTP+CaCBT.

### Interventions

**Figure 1:**
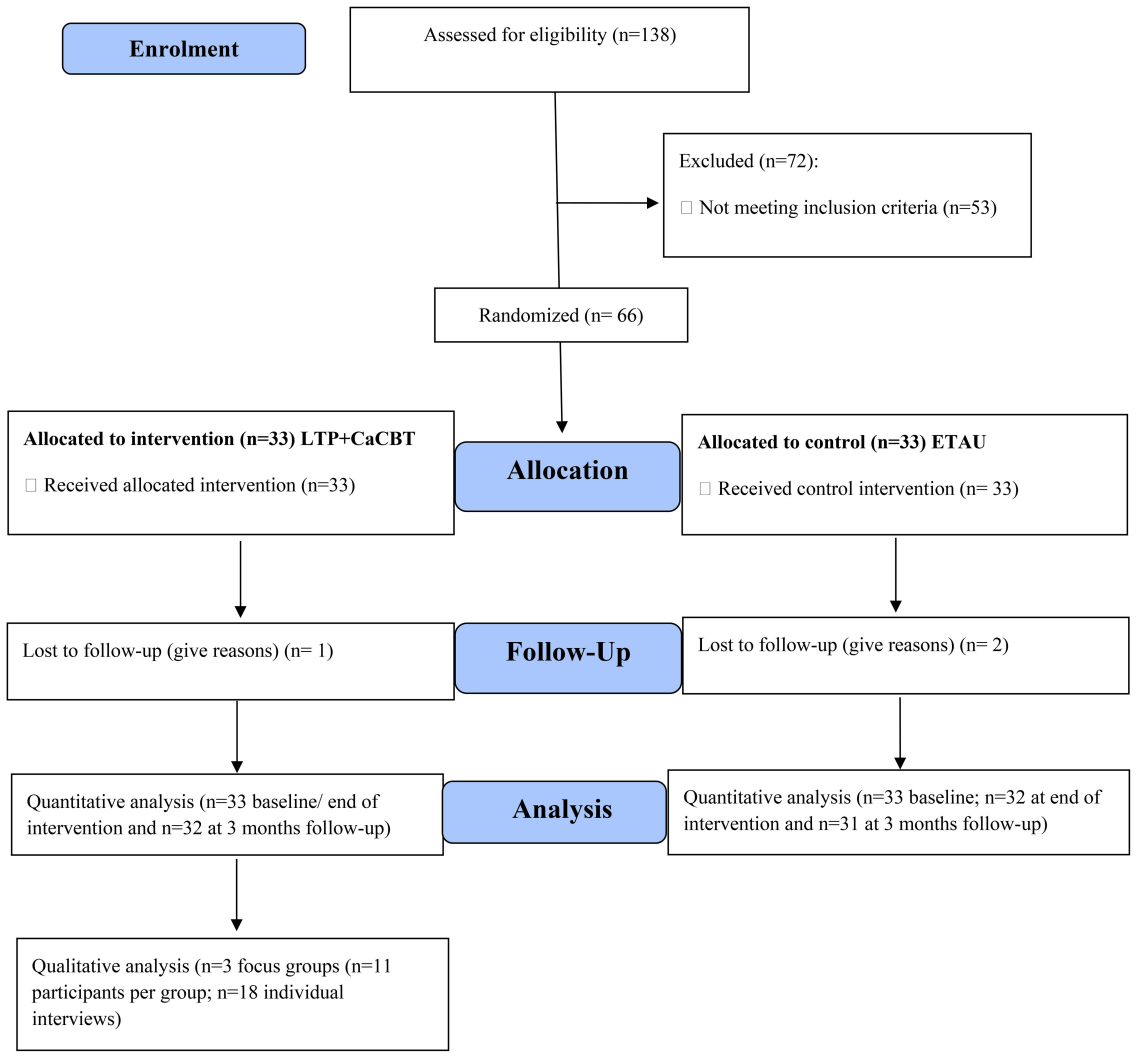
CONSORT Flow Diagram.

The eligible participants (n=66) were recruited (see **Figure 1**), and each was randomly assigned to one of two groups: Group 1: Experimental n=33 received LTP+CaCBT, consisting of 12-group sessions lasted 90 minutes each per week with 10-11 mother-child pairs per sub-group. Group 2: Controlled n=33 received the Enhanced Treatment As Usual (ETAU). For the experimental group, the 12 sessions are the standard recommended duration for brief psychological interventions (35). Due to LTP+CaCBT being manualised and the gender-sensitive nature of the interventions, sessions were delivered by trained female Community Health Workers (CHWs) supported by clinical researchers and supervised by the Principal Investigator and an experienced, trained Community Health Worker every week. One dedicated CHW and clinical researcher facilitated one of the three sub-groups (one centre) of LTP+CaCBT intervention to avoid contamination.

The ETAU group received the participating services’ regular treatment routine. This standard patient care pathway includes service providers’ routine assessment, management, and antidepressant prescriptions. However, it was enhanced because all participants received additional bi-weekly clinical evaluations by psychiatrists and clinical psychologists to ensure their postnatal depression did not deteriorate during the study period.

### Community health workers training

The study’s CHWs were graduate-level non-mental health experts who received (a) two days (24 hours) of extensive trainer training on LTP+CaCBT and (b) weekly one-hour supervision sessions prior to delivering the intervention. The use of CHWs complements the World Health Organisation’s (36) recommendations for task-shifting strategies to use non-mental health professionals to tackle shortages of culturally knowledgeable mental health professionals in low-resource settings such as Nigeria. Das et al. (37) acknowledge the need for task shifting to transfer certain services from high-skill base providers to those with fewer skills or qualifications in healthcare settings affected by chronic workforce shortages.

### Data analyses

#### Quantitative arm

Collated data from the n=66 participants were analysed to determine the study’s primary outcome measures of the LTP+CaCBT intervention’s cultural appropriateness and acceptability using participants’ satisfaction with the intervention (28). For secondary outcome measures, an inferential statistic using the Wilcoxon sign rank test of analysis was conducted to determine the intervention’s preliminary clinical effectiveness in treating postnatal depression and anxiety (29, 30). This nonparametric statistical model was most appropriate for the collated pilot data set (34).

#### Qualitative arm

Three focus groups comprising n=11 participants per group lasting approximately 80 minutes each and n=18 individual interviews lasting about 50 minutes each were conducted with the LTP+CaCBT intervention groups. Participants were recruited into the focus and individual interviews through purposive and opportunity sample techniques. The interviews and focus groups were conducted in person by MSc level clinical researchers (CF; SBM & JEJ) and recorded using Microsoft Teams with recording devices connected to the internet. All interviews were recorded, transcribed verbatim and analysed using an interpretative phenomenological analysis (IPA). The rationale for including a focus group is based on the intervention design, which was delivered in groups with participants sharing lived experiences of postnatal depression. This enabled us to explore the lived experiences both as a group and as individuals. The interview and focus group data reflected the depth and ideographic details reflected in each participant’s account. This analytical lens represents the most appropriate qualitative method for examining participants’ lived experiences of their postnatal depression and engagement with the LTP+CaCBT interventions. All participants’ identifiable information, such as their names in individual interviews and focus group discussions, were anonymised to retain the IPA ideographic nuances in the dataset.

The findings section integrates relevant data verbatim from the transcripts to support themes identified. IPA theoretical features, including phenomenology, single- and double-hermeneutics and idiographic (38, 39), underpinned the data analysis, analytical commentaries and the interpretations of findings. The study’s qualitative arm helped capture rich datasets associated with participants’ experiences of the intervention and postnatal depression from their defined perspectives in ways that could not have been possible using structured psychometric tools or questionnaires (40). All participants’ names, including identifiable information, were anonymised and securely deleted.

The study’s reflexive measures to enhance the study’s fidelity of the qualitative arm’s fidelity. The researchers acknowledged their role in knowledge production from an IPA theoretical lens. Thus, potential researchers’ biases in the study design, data collection, analysis, and interpretation were minimised by ensuring the participants took the lead in recounting their lived experiences related to postnatal depression and engagement with the intervention.

## Results

As shown in **Table 1**, the study observed high attendance, participation and retention rates of 94% across the 12 LTP+CaCBT sessions. The rationale for the high retention of participants in the LTP+CaCBT group could be due to the cultural relevance of the sessions, which drew on content including context-specific scenarios the participants could easily relate to and the interactive nature of the sessions, including the opportunities for peer support.

**Table 1:** Participants’ attendance in LTP+CaCBT experimental group.

Sessions	LTP+CaCBT *N=33*	
	*n*	%
1	29	88
2	28	85
3	31	94
4	29	88
5	30	91
6	32	97
7	32	97
8	31	94
9	31	94
10	32	97
11	32	97
12	33	100
% ÷ 12 sessions _=_ Total		94

*N =* total number of participants randomised to the intervention arm, *n* = total number of participants who attended each session. The percentage comprises those who attended out of 33 participants in the LTP+CaCBT intervention group.

Based on data from **Table 2**, overall, the experimental group demonstrated a higher level of satisfaction with the acceptability of the intervention at 97% when compared to the control group at 34.4%. The experimental group also showed higher satisfaction with the perceived effectiveness of the intervention, which was 70%, compared to the control group, which was 31.3%. A plausible explanation as to why this may be the case could be attributed to the content of the LTP+CaCBT, which is focused on the mother-child play activities promoting bonding attachment alongside the inclusion of cognitive behaviour therapy, which enabled the mothers to practice effective cognitive behaviour management techniques, aimed at reducing depression and negative thinking. The benefit of the intervention was also reported strongly in the experimental group at 85%, suggesting they will recommend the intervention to others when compared to just 31.3% in the control group (ETAU), indicating similar intentions. At the end of the intervention, all 33 participants in the experimental group completed the survey, while in the control group, 32 completed it with one dropout. Overall, the result indicated that the LTP+CaCBT is acceptable, culturally appropriate and feasible, with a higher retention rate than the enhanced treatment in the usual control group.

**Table 2:** Showing the differences between the acceptability scores of the service satisfaction scale for LTP+CaCBT and Enhanced Treatment As Usual (ETAU) groups at 12 weeks end of the intervention.

S/N	Survey questions	LTP+CaCBT *(Exp) n* =33 %					ETAU *(Control) n* = 32 %				
		0	1	2	3	4	0	1	2	3	4
1.	Please rate your satisfaction on the acceptability of the intervention. 0 = Unacceptable; 1 = Slightly Unacceptable; 2 = Not Sure; 3 = Slightly Acceptable; 4 = Acceptable.				3	97		3.13	22	34.4	34.4
2.	Please rate your satisfaction with the effectiveness of the intervention. 0 = Not at all effective; 1 = Ineffective; 2 = Not sure; 3 = Effective; 4 = Very effective.				30	70		3.13	34.4	16	31.3
3.	How would you rate the quality of the intervention? 0 = Worst approach; 1 = Less Quality; 2 = Not sure; 3 = High Quality; 4 = Best approach.				30	70			59.4	19	22
4.	How would you rate your satisfaction with the intervention? 0 = Not satisfied at all; 1 = Not Satisfied; 2 = Not sure; 3 = Satisfied; 4 = Very satisfied.				33	67			47	25	25
5.	Would you recommend the intervention to others? 0 = Definitely no; 1 = No; 2 = Not sure; 3=Yes; 4 = Definitely yes.				15	85			28.13	47	31.3

The Wilcoxon sign rank test analysis for the experimental group (LTP+CaCBT), as shown in **Tables 3**, **4**, highlights a difference in the level of postnatal depression at baseline when compared to the end of the intervention and three follow-ups. This was denoted in the decrease in depression from baseline (*Md* = 5.00) to end of intervention (*Md* = 3.00) with *p=.*001 and at 3 months follow up at (*Md* = 3.00) with *p=.*001. For the GAD, the finding denotes a statistically significant reduction in anxiety from *Md* = 4.00 at baseline to *Md* = 2.00 with *p*. = 001 at the end of the intervention and *Md* = 2.00 with p. = 001 at three months follow-up. Concerning the level of social support, we found an increase in social support from baseline to *Md* = 1.00 to end of intervention *Md* = 3.00 with *p. =* 001. However, the level of social support dropped to *Md* = 2.00 at three months follow up when compared to the end of the intervention, which was *Md* = 3.00.

**Table 3:** Descriptive table showing medians and categories using Wilcoxon Sign Rank Test scores across time for LTP+CaCBT (experimental group) compared to the ETAU (control) group at baseline, end of intervention and 3 months post-intervention.

	Baseline *Md*	EOI *Md*	*z*	*p*	Baseline *Md*	3MFU *Md*	*Z*	*p*
			Baseline * EOI				Baseline * 3MFU	
**PHQ-9**	5.00	3.00	-5.066	.001	5.00	3.00	-4.935	.001
**GAD-7**	4.00	2.00	-5.015	.001	4.00	2.00	-5.00	.001
**OSSS**	1.00	3.00	-4.594	.001	1.00	2.00	-3.871	.001

PHQ-9, patients health questionnaire; GAD-7, Generalised Anxiety Disorder; OSSS, Oslo Social Support Scale; Md, median; EOI, end of intervention; 3MFU, three months follow up post-intervention."*" refers to the differences between timepoints.

**Table 4:** Descriptive table showing medians and categories using Wilcoxon Sign Rank Test scores across time for Enhanced treatment as usual (Control group) at baseline end of and 3 months post-intervention.

	Baseline *Md*	EOI *Md*	*z*	*p*	Baseline *Md*	3MFU *Md*	*z*	*p*
			Baseline * EOI				Baseline * 3MFU	
**PHQ-9**	5.00	4.00	-3.570	.001	5.00	4.00	-2.556	.001
**GAD-7**	4.00	4.00	-1.414	.157	4.00	4.00	-1.134	.257
**OSSS**	1.00	1.00	-.677	.499	1.00	1.00	-1.213	.225

PHQ-9, patients health questionnaire; GAD-7, Generalised Anxiety Disorder; OSSS, Oslo Social Support Scale; Md, median; EOI, end of intervention; 3MFU, three months follow up post-intervention."*" refers to the differences between timepoints.

For the control group, we observed a slight reduction in the level of depression from *Md* = 5.00 at baseline to *Md* = 4.00 at the end of intervention with *p*. = 001 and at three months, *Md* = 4.00. While there was some reduction in depression when compared to the experimental group, the latter performed better with (*Md* = 5.00) to the end of intervention (*Md* = 3.00). Regarding anxiety, we found no changes in the control group when comparing the baseline data at *Md* = 4.00 with the end of the intervention and three months of follow-up data, which stayed the same at *Md* = 4.00. Again, the experimental group performed better, with a reduction in the level of anxiety at baseline *Md* = 4.00 to the end of the intervention and a three-month follow-up with a score of *Md* = 2.00. Finally, regarding the level of social support, similar findings show no changes from baseline *Md* = 1.00, and the end of the intervention, including three months of follow-up, remains on relatively similar scores at *Md* = 1.00. Compared to the experimental group, the LTP+CaCBT performed better. The qualitative findings below provide insight into why we found more promising and positive outcomes with the LTP+CaCBT than the ETAU group.

### Qualitative findings

The qualitative findings were based on participants’ experiences and perceptions of the LTP+CaCBT intervention. Based on analyses underpinned by IPA, the following main superordinate themes were reported: positive behaviour management, effective mother-child interaction and improved relationships, modification of negative thought processes to positive ones, positive experiences and relationship formation. The themes are analysed and reported with supporting extracts from the interview transcripts of data verbatim to retain their originality.

### Positive behaviour management

A recurrent pattern in the dataset was the perceived positive benefit of the programme in addressing the challenges participants faced, especially concerning their recurrent low mood and anger management skill development. Low mood and uncontrollable anger were seen to affect their relationship with others, including their babies, as issues such as anger, if not carefully managed, could have a detrimental effect on the well-being of the mother-child relationship. Talking about this, one participant said:

*“My general experiences and the [LPT+CaCBT) training has taught me a lot in my personal life and the life of my baby. So, I have really learnt a lot. And then, the material used in the training or the programme it was okay for me. The content, the way, the lectures [CHWs], everything was okay to me. I understood it based on what I was taught. [ … ]. I really enjoyed the LT+CaCBT] program. It was helpful. there’s this part, because I have anger issues. Let me say, I am a person that has anger. Just a little thing, it will get me very angry. You mustn’t do much to get me angry. But with this program, it really helped me on how to control myself whenever I’m about to hit my baby. Because at times I can’t control it. But when I remember what I learned here, then I’ll put it into action. Then I’ll not do that. Honestly, I’ve really learned how to control my anger very well”* (Lyop, Zarmangada).

The preceding extract highlights how the participant had a positive experience with the intervention in ways that positively impacted her health and well-being. Lyop’s acknowledgement of the fact that she is always predisposed to anger with consequent adverse behaviours toward her child and how that made her react harshly affects both her and the baby. Due to her engagement with the intervention, the Lyop’s extract denotes her change in behaviour through understanding the mechanism for anger management. This, in turn, enabled Lyop’s role as a mother to be able to avoid harmful behaviours such as hitting her child. In addition to anger management, other participants highlighted how the programme helps them be more patient in their approach and find time to take care of themselves to enhance their parenting experience with their babies. One woman, whilst commenting on the intervention, said:

*“You know, taking care of a baby. You need to be patient. Yes. Because you’re feeling pressure from so many things, so many things I will be thinking here and here. [ … ]. You need to be patient in taking care of the baby because at times it is not easy. Please, even at night, at times when you are going to sleep, the baby will just be needing breast milk. When the baby is sleeping, also is your time also to rest. You understand you can’t do all the things because now you’re stressing yourself. You’re stressing yourself at the same time you’re stressing the baby”* (Kaneng, Kuru).

Nurturing an infant can be very demanding and distressing due to its fragility and need for optimal care, which can put a significant physical and psycho-emotional burden on the mother. The preceding extract shed more light on Kaneng’s lived experience by emphasising how the intervention was helping in understanding the baby’s needs and how best to exercise patience in caring for the baby and taking into consideration the mother’s needs, including when to get rest. Insufficient rest could have an adverse impact on the mother’s well-being, and this could lead to poor outcomes in the mother-child relationship and the child’s developmental outcomes. In making sense of the positive benefit of the programme on the mother, the ability to understand the various stages of child development coupled with the need for the mother to balance this with her health and well-being was seen to create a good experience for both mother and child.

### Enhanced mother-child interaction and relationship

Postnatal depression could significantly impact the mother-child relationship and lead to a deteriorating connection with their babies. Participants expressed a typical pattern when recounting their lived experiences—the perceived improvement in their relationship with their children. Such improvement was construed from diverse perspectives, including effectively communicating with their child and dealing with their stress. Commenting on the former one, the mother said:

*“The part I love most is the communication section, the way we communicate with our children. Before, I did not use to communicate with my baby, but now I find it so interesting to communicate with her. Apart from the communication section, also the health section, taking good care of the baby and the way I will communicate with her when I am angry, the baby gets affected too, when I’m frowning my face in talking to her or shouting at her, the baby will be afraid, but when I’m happy, smiling or laughing, the baby too will know that everything is fine and okay”* (Nvou, Zarmangada).

Nvou’s extract denotes previous unawareness of the importance of appropriate communication with children, including the significant impact of effective communication in improving the mother-child relationship. Being able to understand the significant importance of communicating effectively was construed from a positive perspective. In a near similar vein, another mother, whilst recounting her experience of the intervention, said:

*“I used to be very overwhelmed. I used to be very stressed. I used to be depressed as in- I used to have thoughts that I cannot control, because of my child, I cannot bounce back. I cannot be of any good, I cannot get my life back because of the child. So it now made me to somehow resent my baby. I was feeling as if he [the child] had just come to my life to stress me. But from the intervention, I have now been able to establish communication between myself and him. And also, I have learned to not be overwhelmed by anything, to make conscious efforts to my positive thinking process, as in- to add, to like conscious efforts, to my day-to-day life and everything. Before, I will not lie, I used to hit my toddler, I used to hit him. But with the program, I have learned to read him and understand him and go down to his level and not try to make him to grow up, or act more than his age”* (Ngo Chuwang, Zarmangada).

The preceding extract highlights how having a baby could have a profound effect on the emotional well-being of mothers. In this instance, Ngo Chuwang’s extract seems to suggest that having a baby rather than being construed as a source of joy for the mother was perceived as a burden, which is characterised by stress and depression. The implication of the preceding findings is that the absence of intervention to address the participant’s situation initially led to an adverse response to the child, such that she resorted to physical mechanisms such as hitting the child as a means of venting her frustration. However, following her engagement with Learning through Play plus Culturally adapted Cognitive Behaviour Therapy training, the mother was able to understand how to manage the stress and depression she experiences and, by extension, develop a better communication mechanism with her child characterised by empathy.

### Modification of negative thought processes

Nurturing negative thoughts could have detrimental effects on feelings and behaviour, especially for mothers experiencing postpartum depression. Being able to manage such thoughts could be quite difficult for mothers. As such, a recurrent theme in the participants’ lived experiences of engaging in the intervention was the ability to manage unhelpful thought processes and ways of modifying such thinking to a more positive one. Talking about this, one participant said:

*“The whole programme became very interesting because when they told me of how I can take care of myself, that I should be thinking healthy towards myself, I should remove negative thoughts, I should replace negative thoughts with good positive thoughts. I was very, very happy because somebody like me, I used to think negatively too much. I used to think negative things too much. But when I came here, they told me to replace it with positive thoughts. I became very happy; the whole thing became very interesting. Even if I’m at home, if I want to think negative thoughts, I will quickly replace it with a positive thought, and it makes it easier for me to live healthy without any problem”* (Kangyang, Zarmangada).

As the extract shows, the participant recounts how learning from the programme was translated to her everyday use through the practical application of the skills in addressing negative thoughts. Terms such as *“I used to think negative too much”* highlight how the mother struggles with the complex issues associated with negative automatic thought processes. Learning to modify such recurrent negative thoughts was considered empowering and helpful in elevating her mood, which could positively affect her relationship with her child and others. Another mother, whilst recounting her experience of the intervention, said:

*“And also, we should try and think healthy. Yes, you understand when you think healthy, your baby grows well, and you will not have much stress of taking care of the people where your baby is held. Yes, the baby would not be crying anyhow, you will see her playing. Yes, or if you leave your child, they at times, you know, you won’t even have people that will help you to carry the baby because the baby is dirty. At least when you keep the baby clean, at least you see, everybody will say hi. Whose baby is this one? Fine, baby. At least people will help you carry the baby. At least it will make you not to think too much like that. And again, at times, the issue of family issue, maybe your in-laws [ … ], you need to forget about that one. Your family, the way your baby will grow is very important to you, and you will not even spend much money because you are not going to the hospital”* (Chundung, Kuru).

The participant in the preceding extract emphasised the positive benefit of thinking healthy in impacting the child’s development and well-being. Poor support with child caring was construed as partly impacted by negative thoughts, which could impact the mother’s ability to take care of her child—by extension, preventing others from actually supporting the child’s well-being and health. By participating in the intervention, the participant could appreciate the role of health thought processes in the mother-child well-being and her relationship with others.

### Positive experience and relationship formation

Participants shared their lived experience of the intervention as one characterised by positivity and life-changing skills. The perceived positive ambit of the intervention was construed from diverse perspectives, including the ability to modify negative behaviour, being aware of postpartum depression and being open-minded to help-seeking, including building relationships with others for support. Commenting on the former one, the mother in the focus group said:

*“During the program, I learnt how to think. just like I was being taught how to think, and how I will change my negative thoughts to positive ones. During the lesson, I learned how to change my negative thoughts, and it’s really helping me every day. But I was really going through a lot when this programme started; I was even thinking about negative things. But this programme has helped me, and it also taught me how not to keep quiet. Say something. I need to look for like, a friend to share my problem with and that it is helpful. So, I really like this about the program. It’s really helping me a lot”* (Gyengha, Bukuru).

Being able to seek help through speaking with a confidant and significant others was considered one of the positive experiences Gyangha shared regarding their experiences of engaging with the programme. Another participating mother said:

*“The [LTP+CaCBT] training was generally interesting because it made me- it created an awareness to me that I or we on this side of the country, as in Nigeria precisely, we need psychological intervention, and it’s created an open-mindedness that there’s something called postnatal depression, and we should learn to accept this and learn to find help when needed. And that, um, delivery, giving birth is not just about having, having children is not just about just delivering the baby and living your life. There’s more to it, so the programme opened my mind to the fact that I don’t want my children to grow as broken humans.*

*The programme has helped me because if a mother is broken, there’s no way you can have sound children. That’s why we have like adults these days in society that have, uh! low self-esteem. [ … ] it has shown us that, yes, we should learn to differentiate between psychological problems and spiritual problems because most times before now, anything you are, you can be stressed up and you now feel like you’re not praying enough, or you look for one pastor. But it has actually opened my eyes to know that there’s something called postnatal depression”* (Badung, Bukuru Express).

The extract above highlights the depth of knowledge the participant recounted based on her experience of engaging with the intervention. Terms such as “postnatal depression” are often taken for granted by mothers. Badung’s extract highlights such poor awareness of postnatal depression, which could have a detrimental effect on the mother and, by extension, her child’s upbringing. Therefore, engaging in the intervention was seen as an enlightening platform within which the participant understood her emotions and how best to deal with them. It also highlights the perceived need to seek help when undergoing complex postpartum emotions such as depression rather than merely attribute it to spiritual issues. In essence, the intervention was construed to be highly beneficial in improving the mother’s awareness of depression as a problem not to shy away from and as one where help is worth seeking.

## Discussion and conclusion

The primary aim of this study was to examine the cultural appropriateness, feasibility and acceptability of LTP+CaCBT intervention for treating postnatal depression among Nigerian mothers of children (ages 0-3). Based on the primary outcomes, quantitative findings denoted higher satisfaction in the LTP+CaCBT (experimental group) compared to the enhanced treatment as usual (control group). The LTP+CaCBT also reported higher participant retention engagement and preliminary clinical effectiveness. The preceding findings imply that the LTP+CaCBT is culturally appropriate and acceptable for treating postnatal depression when compared to the enhanced treatment as usual for mothers suffering from postnatal depression in Jos, Nigeria. The reason for the differences could be attributed to the robust design of the LTP+CaCBT intervention’s activities, including the cultural adaptation of the intervention depicting Nigerian culture and context to ensure it helps resonate with the cultural values and needs of the participants. It is also partly attributed to the practical activities, engagement, and treatment outcomes that improve the mother-child well-being.

The cultural appropriateness of LTP+CaCBT for treating postnatal depression in Nigeria is consistent with previous similar studies conducted with African/Caribbean mothers (17). For example, in a study conducted with 19 mothers in Kenya, LTP+CaCBT was found to be culturally appropriate and acceptable for treating postnatal depression in low-income, rural and socio-economically disadvantaged women (19). It is crucial to note that these studies were not fully powered; however, they showed promising results on the potential of LTP+CaCBT in addressing postnatal depression among the identified population. It is also pertinent to note that the present study compared LTP+CaCBT with ETAU, which differs from Jidong et al. (17), which compared LTP+CaCBT with Psychoeducation. However, both studies showed consistency in LTP+CaCBT’s acceptability and cultural appropriateness for treating postnatal depression in Nigeria, Kenya and British African-Caribbean mothers.

Furthermore, in a fully powered study with South Asian women, Husain et al. (20, 24) evaluated the efficacy of learning through play plus (LTP+) intervention to reduce postnatal depression in women with malnourished children in Pakistan. Findings from Husain et al. (24) showed that mother-child attachment and play activities helped improve postnatal mood and reduce depression. The study also found a significant improvement in the quality of the child’s well-being and the mother’s life quality. The present study indicates the significant relevance of cultural adaptations for improving postnatal mental health in collectivist cultures of African, Caribbean, and Pakistani communities. Thus, in integrating collectivist cultural values and cultural practices, the LTP+CaCBT highlight significant benefits to the affected population.

Furthermore, the findings from the study’s secondary outcome measures denote higher reductions in postnatal depression and anxiety with increased social support networks in the LTP+CaCBT group compared to the enhanced treatment as usual group. Insights from the qualitative findings further found that the LTP+CaCBT intervention provided the mothers with practical knowledge of parenting and awareness of mechanisms for behaviour modification, all of which were informed by the theory of attachment (41) and integration of healthy thinking patterns underpinned by the theory of cognitivism (42).

### Limitations and recommendations for future studies

One of the limitations of the present study is the small sample size (n=66), which was not statistically powered to detect the LTP+CaCBT clinical efficacy and effectiveness for treating postnatal depression. As such, it is recommended that future studies conduct fully powered trials comparing LTP+CaCBT with the Treatment As Usual (TAU), including examining the policy implications of the intervention. Another major limitation is the specific focus on one state in Nigeria, and the study acknowledges that Nigeria is a multi-ethnic and cultural country. Future studies are recommended to conduct fully powered trials across Nigeria’s six geopolitical zones while considering cultural diversity. Although, the current study did not analyse the severity of postnatal depression in individual participants or their use of antidepressant medication in both groups. However, future trials may benefit from evaluating these as individual definitive outcome measures. Other useful participants’ social demographics, such as the presence of a supportive partner and/or extended family and any other psychosocial stressors, were not covered in this trial—it may be useful for future studies to examine this information to explore their potential role in predisposing, perpetuating or ameliorating postnatal depression in Nigerian women.

### Study’s contribution and clinical implication

Postnatal depression is a significant global health challenge leading to intergenerational mental health disease burden in women and children. This study’s intervention has made an original contribution through testing the intervention on mothers suffering from postpartum depression in Jos, Nigeria. By extension, contributing to mitigating the disease burden of postnatal depression and further reducing potential child-related disorders in the cohort of mother-child pair beneficiaries in Jos, Nigeria. The LTP+CaCBT intervention also contributed towards the United Nations (40) Sustainable Development Goals (SDGs), such as SDG-3 – which is geared at evidence-based insights into treating postnatal depression in mothers in countries like Nigeria with poor access to culturally relevant care; SDG-4/10 – focusing on addressing inequality and inequity in the current state of postnatal mental health provision.

## Conclusion

Postnatal depression could have a significant adverse negative effect on the mother-child relationship. Such effect spans from poor child development to health challenges and mothers’ inability to successfully provide the required needs to ensure optimal well-being due to experiences of depression. In Nigeria, access to culturally appropriate care is scarce and further impacted by poor socio-economic conditions, stigma, poor awareness of mental health services and religious beliefs. The LTP+CaCBT intervention is promising and has shown to be culturally appropriate, acceptable and helpful in reducing depression. The qualitative dataset of mothers’ intervention experiences also showed positive behaviour management, effective mother-child interaction and relationship, modification of negative thought processes, positive experience and relationship formation. In essence, a fully powered RCT is recommended to evaluate the clinical and cost-effectiveness of LTP+CaCBT, including children’s outcomes compared with the routine treatment as usual.

## Data Availability

The raw data supporting the conclusion of this article will not be available a protect participant confidentiality and privacy.
